# The prevalence of hypertension among Malaysian adults and its associated risk factors: data from Malaysian Community Salt Study (MyCoSS)

**DOI:** 10.1186/s41043-021-00237-y

**Published:** 2021-05-31

**Authors:** Nor Azian Mohd Zaki, Rashidah Ambak, Fatimah Othman, Norazizah Ibrahim Wong, Cheong Siew Man, Mohamad Faizul Azham Morad, Feng J. He, Graham MacGregor, Lalitha Palaniveloo, Azli Baharudin

**Affiliations:** 1 Sarawak General Hospital, Ministry of Health, Kuching, Sarawak Malaysia; 2 Institute for Public Health, National Institutes of Health, Ministry of Health Malaysia, Shah Alam, Selangor Malaysia; 3 Wolfson Institute of Preventive Medicine, Barts and The London School of Medicine & Dentistry, Queen Mary University of London, London, UK

**Keywords:** Malaysian community salt study (MyCoSS), Hypertension, Blood pressure, Cardiovascular disease

## Abstract

**Background:**

Hypertension is one of the most common risk factors for cardiovascular disease and leading cause of mortality globally. The aims of this study were to assess the prevalence of hypertension and its associated risk factors among Malaysian population using data from the Malaysian Community Salt Study (MyCoSS).

**Methods:**

This study was a cross-sectional study using multi-stage stratified sampling method. Data collection was carried out via face-to-face interview at the respondent’s home from October 2017 until March 2018. A total of 1047 respondents aged 18 years and above completed the questionnaires and blood pressure measurement. A person who reported diagnosis of hypertension by a physician and had systolic blood pressure ≥140 mmHg and/or diastolic blood pressure ≥90 mmHg on three readings was categorised as hypertensive. Risk factors of hypertension were analysed using multiple logistic regression.

**Results:**

The prevalence of hypertension in the present study was 49.39% (95% CI 44.27–54.51). There was no statistically significant difference in gender. Age, household income, BMI, and diabetes were significantly associated with hypertension. Hypertension found had inverse association with the level of education. Age was the strongest predictor of hypertension (35–44 years old; OR=2.39, 95% CI=1.39–4.09, 45–54 years old; OR=5.50, 95% CI=3.23–9.38, 55–64 years old OR=13.56, 95% CI=7.77–23.64 and 65 years old and above; OR=25.28, 95% CI=13.33–48.66). Those who had higher BMI more likely to be hypertensive as compared to respondents with normal weight (overweight, OR=1.84; 95% CI=1.18–2.86; obese, OR=4.29% CI=2.56–7.29).

**Conclusion:**

The findings showed that hypertension is prevalent among adults in Malaysia. Those with older age, higher BMI, and diabetes are more likely to have hypertension. Efforts regarding lifestyle modification and education could be important in hypertension management and prevention.

## Background

Cardiovascular disease (CVD) is the largest cause of non-communicable disease (NCD) deaths with an estimated 17.6 million people died worldwide in 2012 [1]. Hypertension is well established as an important risk factor for CVD and was diagnosed as systolic blood pressure (SBP) ≥ 140 mmHg and/or diastolic blood pressure (DBP) ≥90 mmHg [2, 3]. Besides hypertension, CVD risk factors also include abdominal obesity, dyslipidemia, and insulin resistance also known as “metabolic syndrome” [3].

Hypertension has become more prevalent over the past decades worldwide including in developing countries due to various factors, including economic development and population ageing [4]. Data from the National Health and Nutrition Examination Survey (NHANES) found that rates of hypertension among US adults increased from 23.9% in 1988–1994 to 29.0% in 2007–2008 [5]. Prevalence of hypertension was about 27% in Southeast Asian region [6]. In 2010, it is estimated that 325 million Chinese adult aged 18 years or older were diagnosed with hypertension [7]. In Thailand, one out of four Thais had hypertension and almost half of them were not aware that they had high blood pressure [8]. The national prevalence of hypertension among Malaysian adults was 30.3% with rates increasing with age as reported by National Health and Morbidity Survey (NHMS) in 2015 [9].

Many epidemiological studies have established the risk factors for hypertension. These included age, gender, weight, body mass index (BMI), waist circumference (WC), sedentary lifestyle, smoking and alcohol intake [10–12]. Others have implicated lipid disorders, diabetes, salt intake and family history of high blood pressure [13]. More recently, attention has been drawn to excess intake of salt and its major role in the pathogenesis of higher blood pressure [14]. Although local studies on lifestyle risk factors and hypertension were available, the relationship between hypertension and dietary sodium intake are limited. Therefore, this study aims to determine the prevalence and associated risk factors of hypertension including dietary sodium intake among Malaysian adult population using data from the Malaysian Community Salt Study (MyCoSS).

## Methodology

### Study design

MyCoSS is a cross-sectional study which used a multi-stage stratified sample design to obtain a nationally representative sample. One eligible adult aged 18 to 59 years old was selected from each household using Kish table to participate in the survey. A total of 1047 respondents were giving consents to take part in this study. Face-to-face interviewed at respondents’ homes were done using mobile devices during the data collection period between October 2017 and March 2018.

Information on sociodemographic characteristics including gender, age, education level, marital status and household income were collected. Lifestyle behaviours, such as current smoking status and physical activity status, were also assessed. To assess physical activity, a validated Short-International Physical Activity Questionnaire (Short-IPAQ) in Malay version was used [15]. Respondents were asked whether they currently smoked cigarettes to assess current smoking status. Diabetes was diagnosed as random blood sugar ≥11.1 mmol/l or self-report of previous diagnosis of diabetes by doctors [16]. Assessment of dietary sodium intake using food frequency questionnaire (FFQ) from a previous study comprises 104 food items [17].

### Blood pressure measurements

Blood pressure measurements were measured with a digital blood pressure monitor (Omron HBP-1300). Measurement of blood pressure was taken three times in a stable condition with 1- to 2-min interval between each measurement. The average of last two measurement of systolic blood pressure (SBP) and diastolic blood pressure (DBP) were used to determine high blood pressure. The Malaysia Guideline on the Management of Hypertension, 4th Edition, 2013, was used to defined hypertension [18]. A person who reported diagnosis of hypertension previously by a physician and had SBP ≥140 mmHg and/or DBP ≥90 mmHg was diagnosed as hypertensive. Respondents within normal or pre-hypertensive range were categorised into the normal group (normotensive).

### Anthropometry variables

Body weight and height was measured using digital weighing scale (TANITA HD-319) and SECA 213 portable stadiometer with an accuracy of 0.1 kg/0.1 cm, respectively. SECA measuring tape (SECA, Germany) used to measured waist circumference. All measurements were done using validated and calibrated instruments and following standardised protocols. Body mass index (BMI) was computed as weight to the square of height (kg/m^2^) and categorised according to WHO 1998 guidelines [19].

### Statistical analysis

All categorical variables were presented as frequency and percentages while continuous variable was presented as mean with 95% confidence interval (CI). Differences in proportions between categorical variables were analysed using chi-square. Univariate and multiple logistic regression was performed to explore the effects of age, gender, marital status, education level, household income, smoking, physical activity status, diabetes, BMI, and waist circumference as the risk factors for hypertension. The adjusted odds ratios were presented with 95% confidence interval (CI) with *p* value <0.05 was accepted as statistically significant. Data analyses were done using SPSS version 21.0.

## Results

A total of 1047 respondents with mean age of 48.80 years (95% CI 47.03–50.61). The characteristics related with normotensive and hypertensive state among respondents are shown in Table 1. The prevalence of hypertension in present study was 49.39% (95% CI 44.27–54.51) with no significant difference in gender. Prevalence of hypertension was higher among married group (79.96%; 95% CI 74.70–84.36) and those in low household income group (50.83%; 95% CI 43.92–57.70). Higher prevalence of hypertension was shown among those with primary education level (33.77%; 95% CI 27.40–40.78) and secondary education level (48.02%; 95% CI 43.16–52.92) as compared to those with tertiary education (18.20%; 95% CI 12.37–25.96). Individuals with hypertension had significantly greater mean BMI (27.65 kg/m^2^, 95% CI 26.99–28.30, vs 25.68 kg/m^2^, 95% CI 25.08–26.28) and waist circumference index (93.88 cm, 95% CI 92.43–95.32, vs 86.63 cm, 95% CI 85.20–88.06) than those with normal blood pressure.

**Table 1:** Baseline characteristic of respondents with normotensive and hypertension

Characteristic		Normotensive		Hypertensive		*P* value
		%	95% CI	%	95% CI	
Overall		50.60	45.48–55.71	49.39	44.27–54.51	
Sex	Male	50.42	44.74–56.10	49.95	43.77–56.13	1.00
	Female	49.57	43.89–55.25	50.04	43.86–56.22	
Age, years (mean, 95% CI)		40.76	38.92–42.60	57.09	55.62–58.56	<0.001
Age group	<34	38.62	32.83–44.76	4.88	3.03–7.75	<0.001
	35–44	23.41	18.49–29.16	10.84	7.56–15.30	
	45–54	20.40	16.04–25.59	20.20	16.31–24.73	
	55–64	12.62	8.61–18.12	34.29	28.96–40.04	
	>65	4.93	2.99–8.02	29.78	24.28–35.93	
Academic level	None/primary	14.86	10.71–20.24	33.77	27.40–40.78	<0.001
	Secondary	54.58	48.57–60.46	48.02	43.16–52.92	
	Tertiary	30.55	24.61–37.21	18.20	12.37–25.96	
Household income	<RM1999	35.04	27.68–43.20	50.83	43.92–57.70	<0.001
	RM2000–RM3999	30.83	24.78–37.63	28.97	23.13–35.61	
	>RM4000	34.11	26.72–42.36	20.18	14.01–28.19	
Marital status	Single /widowed	28.81	24.07–34.06	20.03	15.63–25.29	0.052
	Married	71.18	65.93–75.92	79.96	74.70–84.36	
BMI (kg/m^2^), (mean, 95% CI)		25.68	25.08–26.28	27.65	26.99–28.30	<0.001
BMI (kg/m^2^)	<25	46.06	40.18–52.06	31.63	26.18–37.64	<0.001
	25–30	36.93	32.08–42.06	38.34	32.14–44.92	
	≥ 30	16.99	13.25–21.53	30.01	24.39–36.43	
WC, cm (mean, 95% CI)		86.63	85.20–88.06	93.88	92.43–95.32	<0.001
WC, cm						
Men <90, Women <80	No	41.82	36.72–47.09	23.20	18.15–29.17	<0.001
Men ≥90, Women ≥80	Yes	58.17	52.90–63.26	76.79	70.82–81.84	
Smoking status	No	74.50	69.43–70.99	82.53	77.00–86.95	<0.001
	Yes	25.49	21.00–30.56	17.46	13.04–22.99	
Physical activity	Inactive	42.93	35.61–50.57	40.53	34.33–47.04	0.448
	Active	57.06	49.42–38.59	59.46	52.95–65.66	
Diabetes	No	90.81	85.42–94.34	64.44	58.36–70.09	<0.001
	Yes	9.18	5.65–14.57	35.55	29.90–41.63	
Sodium intake (mg/day)	< 2000	39.56	33.49–45.93	52.47	47.35–57.54	<0.001
	≥ 2000	60.43	54.03–66.50	47.52	42.45–52.64	

Table 2 shows various risk factors associated with hypertension. Univariate analysis showed that age, academic level, household income, marital status, BMI, waist circumference, current smoking, diabetes and total sodium intake were independently associated with increased odds for hypertension. Hypertension shown had negatively associated with the level of education. After controlling for other covariates, age was the strongest predictor of hypertension (35–44 years old; OR=2.39, 95% CI=1.39–4.09, 45–54 years old; OR=5.50, 95% CI=3.23–9.38, 55–64 years old OR=13.56, 95% CI=7.77–23.64 and 65 years old and above; OR=25.28, 95% CI=13.33–48.66). Overweight or obese respondents were more likely to be hypertensive compared to normal weight (overweight, OR=1.84; 95% CI=1.18–2.86; obese, OR=4.29; 95% CI=2.56–7.29).

**Table 2:** Factors associated with hypertension from univariate and multivariate logistic regression model

Characteristic		Univariate analysis		Multivariate analysis	
		OR (95% CI)	*p* value	OR (95% CI)	*p* value
Sex	Male	1 .00 (reference)	0.986		
	Female	1.02 (0.78–1.28)			
Age group	>34	1 .00 (reference)		1 .00 (reference)	
	35–44	3.34 (2.04–5.45)	<0.001	2.39 (1.39–4.09)	0.002
	45–54	7.35 (4.62–11.67)	<0.001	5.50 (3.23–9.38)	<0.001
	55–64	19.35 (12.01–31.17)	<0.001	13.56 (7.77–23.64)	<0.001
	>65	32.04 (18.58–55.25	<0.001	25.38 (13.13–48.66)	<0.001
Academic level	None/primary	1 .00 (reference)		1 .00 (reference)	
	Secondary	0.37 (0.28–0.51)	<0.001	0.69 (0.46–1.02)	0.064
	Tertiary	0.19 (0.14–0.29)	<0.001	0.58 (0.34–0.98)	0.042
Household income	<RM1999	1 .00 (reference)		1 .00 (reference)	
	RM2000–RM3999	0.65 (0.49–0.87)	0.003	1.54 (1.04–2.28)	0.031
	>RM4000	0.43 (0.31–0.59)	<0.001	0.74 (0.46–1.16)	0.735
Marital status	Single/widowed	1 .00 (reference)		1 .00 (reference)	
	Married	1.32 (1.01–1.74)	0.045	1.12 (0.47–1.61)	0.550
BMI (kg/m^2^)	<25	1 .00 (reference)		1 .00 (reference)	
	25–30	1.85 (1.39–2.46)	<0.001	1.84 (1.18–2.86)	0.007
	≥ 30	3.34 (2.40–4.64)	<0.001	4.29 (2.55–7.29)	<0.001
WC, cm					
Men <90, women <80	No	1 .00 (reference)		1 .00 (reference)	
Men ≥90, women ≥80	Yes	2.53 (1.94–3.30)	<0.001	1.16 (0.74–1.79)	0.518
Smoking status	No	1 .00 (reference)	0.003	1 .00 (reference)	0.82
	Yes	0.63 (0.46–0.86)		0.96 (0.64–1.42)	
Physical activity	Inactive	1 .00 (reference)	0.438		
	Active	0.91 (0.71–1.16)			
Diabetes	No	1 .00 (reference)	<0.001	1 .00 (reference)	<0.001
	Yes	5.71 (3.89–8.39)		2.48 (1.59–3.87)	
Sodium intake (mg/d)	< 2000	1 .00 (reference)	<0.001	1 .00 (reference)	0.686
	≥ 2000	0.53 (0.42–0.68)		0.94 (0.68–1.29)	

*Significance at *p* value < 0.05

## Discussion

The prevalence of hypertension in our study was 49.4%, which is higher than the nationally representative data set of 19,936 Malaysian adults in 2015 (35.3%) [20]. However, the result was comparable with Abdul-Razak et al. who found 47.9% of Malaysian adults aged more than 30 years were diagnosed with hypertension [21]. The rising of hypertension and other non-communicable disease (NCD) was correlated with modernisation and an increasing economy status with most Malaysians adopted sedentary lifestyles and high consumption of high calorie, salty and fatty food [22].

This study found no significant difference in prevalence of hypertension by gender. This finding was in contrast with findings from other studies that showed a higher prevalence of hypertension was detected in men than in women [21]. Limited sample size may have influenced to the differing results in rate of hypertension among men and women. Consistent with other studies, hypertension prevalence was lowest among those with higher income status and higher academic level among the Malaysian population, similar to other countries in the region [23, 24].

Multivariate analysis showed that age, higher academic level, moderate household income, BMI and diabetes were significantly associated with hypertension. This study found that the prevalence of hypertension increased with age in both men and women. Similar results were seen among adults in India and Taiwan [11, 25]. Academic level was shown as a protective factor and the result was similar with a study in China [13]. Better knowledge on healthy lifestyle and being well informed about blood pressure management may contribute to lower risk of hypertension among those with higher education level [26].

Our findings confirmed the finding from previous studies that BMI and diabetes were associated with high risk of hypertension [7, 27]. A study conducted by Papathanasiou et al. suggest greater BMI was significantly and directly related with increased resting blood pressure in both gender [28]. However, waist circumference did not show an association with hypertension in this study. Feng et al. suggested that waist circumference is strongly independently associated with diabetes because the measurement reflects an accumulation of intra-abdominal fat that could lead to insulin resistance. Meanwhile, BMI is associated with hypertension due to increase in body weight (BMI) which may increase body fluid volume, peripheral resistance and cardiac output [25].

Excess consumption of dietary sodium has a main role in the pathogenesis of elevated blood pressure. A study by Zhang et al. showed that high sodium intake was positively related with systolic blood pressure [26]. Our result indicated that in multivariable analysis, no significantly different between sodium intake and hypertension. The results may be due to limitations of cross-sectional study design and the dietary data that was collected using food frequency questionnaire. Compared with 24-h sodium excretion, estimation participants’ usual sodium intake from food frequency questionnaire in this study may under or overestimate actual intake. Other possible reason was that respondents may already be aware about risk of high salt diet with hypertension. Study by Abdul-Razak et al. found that awareness of hypertension among Malaysian adult more than 30 years of age was 53.2 % [21].

There are several strengths in our study: first, the random selection of the respondents, sample size, and high participation rate, and second, examination of the relationship between BP and anthropometry, lifestyle risk factors, and dietary sodium intake added the strength of this study. The main limitation in this study was that the sampling framework is not the total population representative, and therefore, caution is needed in extrapolating the information. The limitation of sampling size may have overestimated the prevalence of hypertension as compared with national prevalence. Nevertheless, associated risk factors of hypertension indicated in this study were in line with other studies except for gender.

## Conclusion

Current finding revealed that hypertension is prevalent among adult in Malaysia and it found that age, BMI, those with middle household income and diabetes were predictors of hypertension. These results further underline the need for routine blood pressure check-up to identify subjects with high-risk of hypertension. Strategies to strengthen the monitoring of modifiable risk factors including optimal weight control should be advocate for better hypertension control in Malaysia.

## Data Availability

The dataset of this article belongs to the MyCoSS project. At present, the data are not publicly available but can be obtained from the authors upon reasonable request and with the permission from the Director General of Health, Malaysia.

## Abbreviations

MyCoSS: Malaysian Community Salt Study
NHMS: National Health and Morbidity Survey
CVD: Cardiovascular disease
NCD: Non-communicable disease
BMI: Body mass index
SBP: Systolic blood pressure
DBP: Diastolic blood pressure
SD: Standard deviation
CI: Confidence interval
